# Activation of Transient Receptor Potential Vanilloid 1 Channels in the Nucleus of the Solitary Tract and Activation of Dynorphin Input to the Median Preoptic Nucleus Contribute to Impaired BAT Thermogenesis in Diet-Induced Obesity

**DOI:** 10.1523/ENEURO.0048-21.2021

**Published:** 2021-04-08

**Authors:** Ellen P. S. Conceição, Christian A. Reynolds, Shaun F. Morrison, Christopher J. Madden

**Affiliations:** 1 Department of Neurological Surgery, Oregon Health & Science University, Portland, OR 97239; 2 Department of Emergency Medicine, Wayne State University, Detroit, MI 48201

**Keywords:** brown adipose, high-fat diet, κ opioid, overweight, sympathetic nerve activity, thermoregulation

## Abstract

The impairment of cold-evoked activation of brown adipose tissue (BAT) in rats fed a high-fat diet (HFD) requires the activity of a vagal afferent to the medial nucleus of the solitary tract (mNTS). We determined the role of transient receptor potential vanilloid 1 (TRPV1) activation in the mNTS, and of a dynorphin input to the median preoptic nucleus (MnPO) in the impaired BAT thermogenic response to cold in HFD-fed rats. The levels of some linoleic acid (LA) metabolites, which can act as endogenous TRPV1 agonists, were elevated in the NTS of HFD rats compared with chow-fed rats. In HFD rats, nanoinjections of the TRPV1 antagonist, capsazepine (CPZ) in the NTS rescued the impaired BAT sympathetic nerve activity (BAT SNA) and thermogenic responses to cold. In contrast, in chow-fed rats, cold-evoked BAT SNA and BAT thermogenesis were not changed by nanoinjections of CPZ into the NTS. Axon terminals of NTS neurons that project to the dorsal lateral parabrachial nucleus (LPBd) were closely apposed to LPBd neurons that project to the MnPO. Many of the neurons in the LPBd that expressed c-fos during cold challenge were dynorphinergic. In HFD rats, nanoinjections of the κ opioid receptor (KOR) antagonist, nor-binaltorphimine (nor-BNI), in the MnPO rescued the impaired BAT SNA and thermogenic responses to cold. These data suggest that HFD increases the content of endogenous ligands of TRPV1 in the NTS, which increases the drive to LPBd neurons that in turn release dynorphin in the MnPO to impair activation of BAT.

## Significance Statement

Obese humans have brown adipose tissue (BAT); however, there has been no explanation for the consistent observation of reduced cold-activated BAT thermogenesis in obese humans. We previously established the high-fat diet (HFD)-fed rat as a good model that mimics the human characteristics of impaired cold activation of BAT during obesity. The data presented here identify necessary components of the central neural circuitry for HFD-induced impairment of BAT activation. These findings contribute substantially to our understanding of both the basic brain circuitry controlling BAT and the effect of a HFD to hijack this circuitry leading to reduced BAT metabolic energy consumption and excess adiposity. These insights should contribute to the discovery of novel therapeutic approaches to promote metabolism of fat.

## Introduction

Obesity contributes significantly to morbidity and mortality. Obesity is characterized by excess energy storage because energy intake is greater than energy expenditure, resulting in adipose tissue hypertrophy and body weight gain. The activity of the sympathetic nerves innervating brown adipose tissue (BAT) leads to the oxidation of fatty acids and the production of heat thereby contributing significantly to the level of BAT metabolism and to whole-body energy expenditure. Importantly, cold-evoked activation of BAT thermogenesis is impaired in obese humans (van Marken Lichtenbelt et al., 2009; Leitner et al., 2017) and rats (Sakaguchi et al., 1989; Madden and Morrison, 2016). Understanding the relationship between impaired BAT metabolic thermogenesis and obesity could reveal novel therapeutic targets to combat metabolic syndrome.

The median preoptic nucleus (MnPO) is an important integrative site for several crucial homeostatic functions, including thermoregulation (McKinley et al., 2015). The MnPO receives inputs from subdivisions of the lateral parabrachial nucleus (LPB). In general, neurons in the external lateral region of the LPB (LPBel) are activated by skin cooling (Bratincsák and Palkovits, 2004; Nakamura and Morrison, 2008b), while neurons in the dorsal region of the LPB (LPBd) are activated by skin warming (Bratincsák and Palkovits, 2004; Nakamura and Morrison, 2010), although there are some neurons in the LPBd that are activated during skin cooling (Nakamura and Morrison, 2008b; Geerling et al., 2016). The MnPO contains both abundant dynorphin immunoreactive fibers, including preprodynorphin (ppDyn) projections from the LPB (Hermanson et al., 1998), and κ opioid receptors (KORs; DePaoli et al., 1994). Dynorphinergic activation of KOR in MnPO is associated with hypoxia-induced inhibition of thermogenesis (Scarpellini Cda et al., 2009). In addition, microdialysis of a KOR agonist in the MnPO induces hypothermia in rats (Xin et al., 1997). A population of dynorphinergic LPBd neurons receive inputs from transient receptor potential vanilloid 1 (TRPV1)-responsive neurons in the medial nucleus of the solitary tract (mNTS; Hermanson and Blomqvist, 1996; Okada et al., 2019). Activation of neurons in the mNTS with the glutamate receptor agonist, NMDA, suppresses BAT sympathetic nerve activity (SNA) and BAT thermogenesis (Cao et al., 2010). Activation of TRPV1 channels within the mNTS, which are located on unmyelinated vagal afferent terminals (Hermes et al., 2016), also inhibits BAT SNA and decreases BAT metabolism (Mohammed et al., 2018). Together, these findings are consistent with a BAT sympathoinhibitory pathway in which TRPV1-expressing neurons in mNTS excite dynorphinergic neurons in LPBd that project to inhibit BAT sympathoexcitatory neurons in the MnPO (da Conceicao et al., 2020). Our data not only provide further support for this inhibitory regulation of BAT energy expenditure driven by an as yet unidentified vagal viscerosensory input to mNTS, but also implicate this pathway in the suppression of BAT thermogenesis and BAT energy expenditure induced by a high-fat diet (HFD).

Interestingly, ∼50 endogenous lipids can regulate TRP channel (including TRPV1) activity in sensory neurons (Taberner et al., 2015). Oxidized metabolites of the essential fatty acid, linoleic acid (LA), including 9- and 13-hydroxyoctadecadienoic acid (HODE), their dihydroxy-metabolites (DiHOMEs), and epoxides of LA (EpOMEs) are among the endogenous ligands of TRPV1 (Patwardhan et al., 2010; Osthues and Sisignano, 2019). Thus, a diet rich in fat with a high content of LA has the potential to influence the level of such TRP ligands in tissues and organs, including the brain.

We investigated the neural circuit underlying one of the obesogenic, metabolic adaptations evoked by HFD feeding in rats: impairment of the sympathetic activation of BAT thermogenesis in diet-induced obesity. We tested the hypotheses that activation of TRPV1 channels in the mNTS and of KORs in the MnPO is necessary for the HFD-induced impairment of cold-evoked sympathetic activation of BAT. We also provide anatomic support for a neural circuit connecting mNTS neurons, via dynorphinergic LPBd neurons, to thermoregulatory neurons in the MnPO, that could underlie the HFD-induced reduction in cold-evoked BAT thermogenesis. Additionally, we evaluated whether HFD alters the availability of TRPV1 ligands in the mNTS, which could contribute to activation of this BAT sympathoinhibitory pathway.

## Materials and Methods

Seventy-two male Sprague–Dawley rats (300–640 g from Charles River Laboratories) were used in these studies. The rats were housed at 22–23°C with a standard 12/12 h light/dark cycle. All procedures conform to the regulations detailed in the National Institutes of Health *Guide for the Care and Use of Laboratory Animals* and were approved by the Animal Care and Use Committee of the Oregon Health and Science University.

In the control group, the rats were fed a chow diet with 13% of fat content from lard (Laboratory Rodent Diet 5001, Lab Supply), while the HFD group received special diet with 45% of fat content from lard and soybean oil (Research Diets D1245) for at least eight weeks, both groups received *ad libitum* access to food and water. Before the experiments, the body composition was measured by Echo-MRI analysis. HFD rats used for the experiments had obtained at least 27% body fat.

### Anatomical tracing studies

For the surgical procedure, rats were anesthetized with isoflurane in 100% O_2_ (4% for induction, 2% for maintenance) and placed in a stereotaxic apparatus (*n* = 4). The rats received Rimadyl (5 mg/kg, s.c.) and penicillin G (40 kIU/kg, i.m.) and were hydrated with isotonic saline (5 ml, s.c.). Unilateral pressure-injections (Picospritzer II, General Valve) of FluorGold (FG; 60 nl at 2% in saline; Fluorochrome Inc.) and biotinylated dextran amines (BDA; 10% in saline, 60 nl, N-7167, Invitrogen Inc.) were made via glass micropipettes (tip inner diameter: 10–20 μm) stereotaxically positioned in the MnPO (at bregma, on the midline, and 6.5–7.0 mm ventral to dura) and in the NTS (0.5 mm rostral to calamus scriptorius, 0.5 mm lateral to the midline, and 0.5 mm ventral to dura), respectively. Each rat recovered for 7 d and received Rimadyl (5 mg/kg, s.c.) for the first 3 d to reduce postsurgical inflammation. On the 10th day, the rats were anesthetized with isoflurane in 100% O_2_ (1.5–2%) and placed at the stereotaxic apparatus and wrapped with a water perfused blanket for 2 h (water temperature: ∼35°C, a cold stimulus that increases BAT SNA and thermogenesis in chow-fed rats; Madden and Morrison, 2016). Then the rats received a large dose of anesthetic (urethane, 750 mg/kg, i.p., together with α-chloralose, 60 mg/kg, i.p.) and were perfused transcardially with 200 ml of a 0.9% sodium chloride solution followed by 250–300 ml of 4% formaldehyde in 0.1 m sodium phosphate buffer (pH 7.4). Subsequently, the brain was removed and postfixed in 4% formaldehyde at 4°C for 2 h and then cryoprotected with 30% sucrose in 10 mm sodium phosphate buffer (pH 7.4) overnight. The brains were cut into 35-μm-thick coronal sections on a freezing-stage microtome.

### Retrograde and anterograde labeling and immunohistochemistry (IHC)

Consecutive sections from a 1:6 set of sections (175 μm between consecutive sections) were processed. The retrograde tracer, FG was identified by autofluorescence. ppDyn or c-Fos were identified by an overnight incubation in a guinea pig-anti-ppDyn antibody (Neuromics, GP10110, used at a 1:1000 dilution) or a rabbit-anti-c-Fos antibody at a 1:10,000 dilution (EMD/Calbiochem #PC38) followed by several rinses in PBS and a 2-h incubation in a goat anti-guinea pig or donkey anti-rabbit immunoglobulin G antibody (conjugated to Alexa Fluor 488 or Alexa Fluor 594, Invitrogen, A11073, A11076, A21206, or A21207 at a 1:500 dilution). The anterograde BDA was identified using streptavidin Alexa Fluor A594 (Invitrogen, S32356, at a 1:500 dilution). After several rinses in PBS, sections were mounted onto gelatin-coated glass slides and coverslipped with Prolong Gold anti-fade reagent (Invitrogen). An Olympus BX51 microscope with appropriate filter sets was used to assess the labeling. Adobe Photoshop was used to adjust brightness and contrast and to assemble the photomicrographs.

### Liquid chromatography-tandem mass spectrometry (LC-MS) analysis

Plasma and brain tissue samples were analyzed for eicosanoid quantification as previously described with minor modifications (Maddipati et al., 2014). Plasma samples (200 μl) were spiked with an internal standard (IS) mix and diluted to 1 ml with 15% methanol in water (*n* = 6 each group). For brain tissue samples, the rats were decapitated and the brain was quickly removed and placed on dry ice, once frozen ∼100-μm coronal tissue sections of each brain region [mNTS and dorsomedial hypothalamus (DMH)] were prepared, the tissue samples were harvested and the samples were frozen at −80°C until further processing. For the mNTS, the tissue samples were taken bilaterally from the mNTS (extending rostrally from calamus scriptorius ∼100 μm, laterally ∼0.5 mm and ∼0.5 mm deep from the dorsal surface). For the DMH, tissue samples were taken bilaterally and included the entire area spanning from the mammillothalamic tract to the fornix and medially to the third ventricle. Brain tissue samples (*n* = 6 each group) were weighed and homogenized in 0.5 ml PBS (pH 7.4), using Zirconium beads on a high‐frequency oscillator (Precellys homogenizer, Bertig Instruments). The homogenates were centrifuged at 10,000 × *g* for 10 min and the supernatant was collected and spiked with the IS mix and diluted to 1 ml with 15% methanol in water Samples were purified on C18 solid-phase extraction cartridges (30 mg sorbent, 1 ml; Strata-X; Phenomenex). The cartridges were preconditioned with 1 ml methanol followed by 1 ml 15% methanol in water. The diluted, IS-spiked samples were applied to the cartridge, washed with 2 ml of 15% methanol and 2 ml hexane, and dried in a vacuum for 30 s. The cartridge was eluted with 0.5 ml methanol containing 0.1% formic acid directly into 1.5 ml LC-MS autosampler vials. The eluate was dried under a gentle stream of nitrogen, and the residue was immediately reconstituted with 25 μl methanol. The reconstituted sample was stored at −80°C until LC-MS analysis. LC-MS analysis was performed using a C18 column [Luna, C18(2); 2.1 × 150 mm, 3 μm; Phenomenex] and QTrap5500 mass analyzer (AB Sciex) in the negative ion mode. Multiple reaction monitoring (MRM) was used to detect unique molecular ion–daughter ion combinations for each analyte. The data were collected with Analyst 1.5.2 software (AB Sciex), and the MRM transition chromatograms were quantitated by MultiQuant software (AB Sciex). The IS signals in each chromatogram were used for normalization, recovery, and relative quantitation of each analyte. The concentration of each detected analyte in plasma samples was expressed as ng per mL, and the concentration of each detected analyte in brain tissue samples was expressed as ng/g of tissue.

### Surgical and experimental procedures for the recording of BAT SNA

The rats were anesthetized with isoflurane (2–3% in 100% O_2_) with the level of anesthesia verified by the lack of motor responses to a strong tail or foot pinch. The femoral artery was cannulated for monitoring mean arterial pressure (MAP) and heart rate (HR). The femoral vein was cannulated for intravenous (4) administration of anesthesia (urethane, 750 mg/kg iv α-chloralose, 60 mg/kg iv; both supplemented at 10% of initial dose per hour, i.v.) and neuromuscular blockade (D-tubocurarine, 0.6 mg per rat, i.v., supplemented with 0.3 mg/h, i.v.). Isoflurane anesthesia was terminated once urethane/α-chloralose anesthesia was established. The adequacy of anesthesia was assessed hourly and verified by the lack of cardiovascular or motor responses to a strong tail pinch before the neuromuscular blockade. The trachea was cannulated for artificial ventilation with 100% O_2_ at a minute volume of 180–240 ml, such that the resting end-expired CO_2_ (Exp CO_2_) remained between 3.5% and 5.0%. The rats were positioned prone in a stereotaxic frame, with the incisor bar at −4 mm below interaural zero. A water-perfused thermal blanket was wrapped completely around the shaved trunk from the shoulders to the hips. Thermocouples (Physitemp) were inserted into the rectum to measure core body temperature (TCORE), into the left interscapular BAT pad to measure BAT temperature (TBAT), and onto the flank skin under the thermal blanket to measure skin temperature (TSKIN; TC-1000 thermocouple reader, Sable Systems). TCORE was maintained at ∼37.0°C with a thermostatically controlled heating lamp, in combination with the water-perfused thermal blanket, except as noted during skin cooling episodes.

### Procedures for free-behaving experiments

Rats were anesthetized with 2% isoflurane in oxygen and implanted with a dual thermistor telemetry transmitter (F40-TT, DSI a division of Harvard Bioscience) for recording TBAT (thermistor placed in the interscapular BAT pad) and core body temperature (by placing the thermistor in the peritoneum). At the end of the surgical procedure, rats received Rimadyl (5 mg/kg, s.c.) and penicillin G (40 kIU/kg, i.m.) and were hydrated with isotonic saline (5 ml, s.c.). Rats recovered for at least 10 d in a temperature-controlled environmental chamber maintained at 24°C. On the day of the experiment, HFD rats received an injection of nor-binaltorphimine (nor-BNI; 10 mg/kg, i.p.) or a sham injection. A chow-fed group of rats received sham injections. Two hours after the injections (or sham), the temperature of the chamber was changed to 15°C for two additional hours, at which time rats were deeply anesthetized with isoflurane (3% in O_2_) followed by urethane/α chloralose (1000 and 80 mg/kg, i.p., respectively), and then exsanguinated.

### Administration of drugs

Drugs were administered into brain sites via stereotaxically positioned, nanoinjection pipettes (20-μm tip diameter) connected to a pneumatic injector (Toohey). Nanoinjections were made in the MnPO (at bregma, on the midline, and 6.5–7.0 mm ventral to dura; and in the mNTS (0.5 mm rostral to calamus scriptorius, 0.5 mm lateral to the midline, and 0.5 mm ventral to dura). The drug nanoinjection volume was 60 nl (estimated using a calibrated microscope reticule to observe the displacement of the fluid meniscus in the glass pipette). We used TRPV-1 antagonist, capsazepine (CPZ; 100 μm; Tocris Bioscience) dissolved in 1% ethanol, and KOR antagonist nor-BNI (27 μm; Sigma-Aldrich) dissolved in isotonic saline. Nanoinjection of the 1% ethanol and the isotonic saline vehicle had no effects on either BAT thermogenic or cardiovascular variables.

### Histologic localization of injection sites

The microinjection sites were marked by pressure nanoinjection of fluorescent polystyrene microspheres (FluoSpheres, F8797, F8801, or F8803; Invitrogen) included in the injectate (1:100 dilution of FluoSpheres). After the physiological recordings, rats were perfused (5% formaldehyde) transcardially, and brains were removed, postfixed (2–12 h), and sectioned on a microtome (60-μm coronal sections). The sections were mounted on slides, and nanoinjection sites were localized and photographed.

### Statistical analysis

For analysis of BAT SNA, a continuous measure (4-s bins) of BAT SNA amplitude was obtained as the root mean square (rms) value of the BAT SNA, calculated (Spike 2, CED) as the square root of the total power in the 0.1- to 20-Hz frequency band of the autospectra of sequential 4-s segments of BAT SNA. To normalize slight differences in nerve recording characteristics among experiments, BAT SNA values are expressed as % of baseline (BL), where the BL value of BAT SNA in each experiment is the minimum rms value of BAT SNA when TCORE and TSKIN are sufficiently warm (>37°C) to eliminate any cold-evoked BAT SNA. Pretreatment (i.e., control) values of BAT SNA (expressed as % BL) and other variables were the mean values during the 120-s period before treatment. Pretreatment control conditions varied depending on the experimental protocol. In some cases, we used a high TSKIN and TCORE (≥37.0°C, warm condition), resulting in low levels of BAT SNA, or alternatively a low TSKIN and TCORE (≤35.0°C, cold condition). The amplitudes of treatment-evoked responses were calculated from the mean levels of the variables during the 30 s period of the peak response occurring within 120 min of the treatment. The data are expressed as mean ± SEM. The effect of the treatment with CPZ or nor-BNI, were analyzed by comparing the prenanoinjection and postnanoinjection values in chow-fed and HFD-fed groups in the cold and warm conditions using paired three-way ANOVA with *post hoc* Sidak’s test. Unpaired one-tailed *t* tests were used for comparisons of preinjection values between the HFD and chow groups. Confidence intervals were generated using Estimation Statistics β (https://www.estimationstats.com; Ho et al., 2019). Two-sided permutation *t* tests were used to compare the eicosanoid levels between the HFD and chow groups. In HFD-fed versus chow-fed rats, the percentage of retrogradely-labled neurons in the LPBd containing cold-evoked c-Fos was compared using a two-sided permutation *t* test; *p* < 0.05 was considered significant. Estimation statistics and two-sided permutation tests (http://www.estimationstats.com) were used to compare the cold-evoked change in TBAT between free-behaving chow-fed, HFD, and HFD rats pretreated with nor-BNI.

## Results

### Blockade of TRPV1 in the NTS recovers the BAT thermogenic response to cooling in HFD rats

Our findings that TRPV1 activation in the mNTS inhibits BAT SNA (Mohammed et al., 2018), and that signaling on vagal afferents, which express TRPV1, is necessary for the impairment of BAT activation during HFD (Madden and Morrison, 2016), led to the hypothesis that activation of TRPV1 in the mNTS during HFD contributes to the impairment of the cold-evoked BAT thermogenesis. The observation that several metabolites of LA, a significant component of the HFD used in our studies, can act as TRPV1 agonists (Patwardhan et al., 2010; Osthues and Sisignano, 2019) also makes this hypothesis particularly attractive. To test this hypothesis, we made bilateral nanoinjections of CPZ, a TRPV1 antagonist, in the mNTS of chow-fed or HFD-fed rats, while their TSKIN and TCORE were maintained at 37°C (warm) or 35°C (cold challenge).

**Figure 1.**
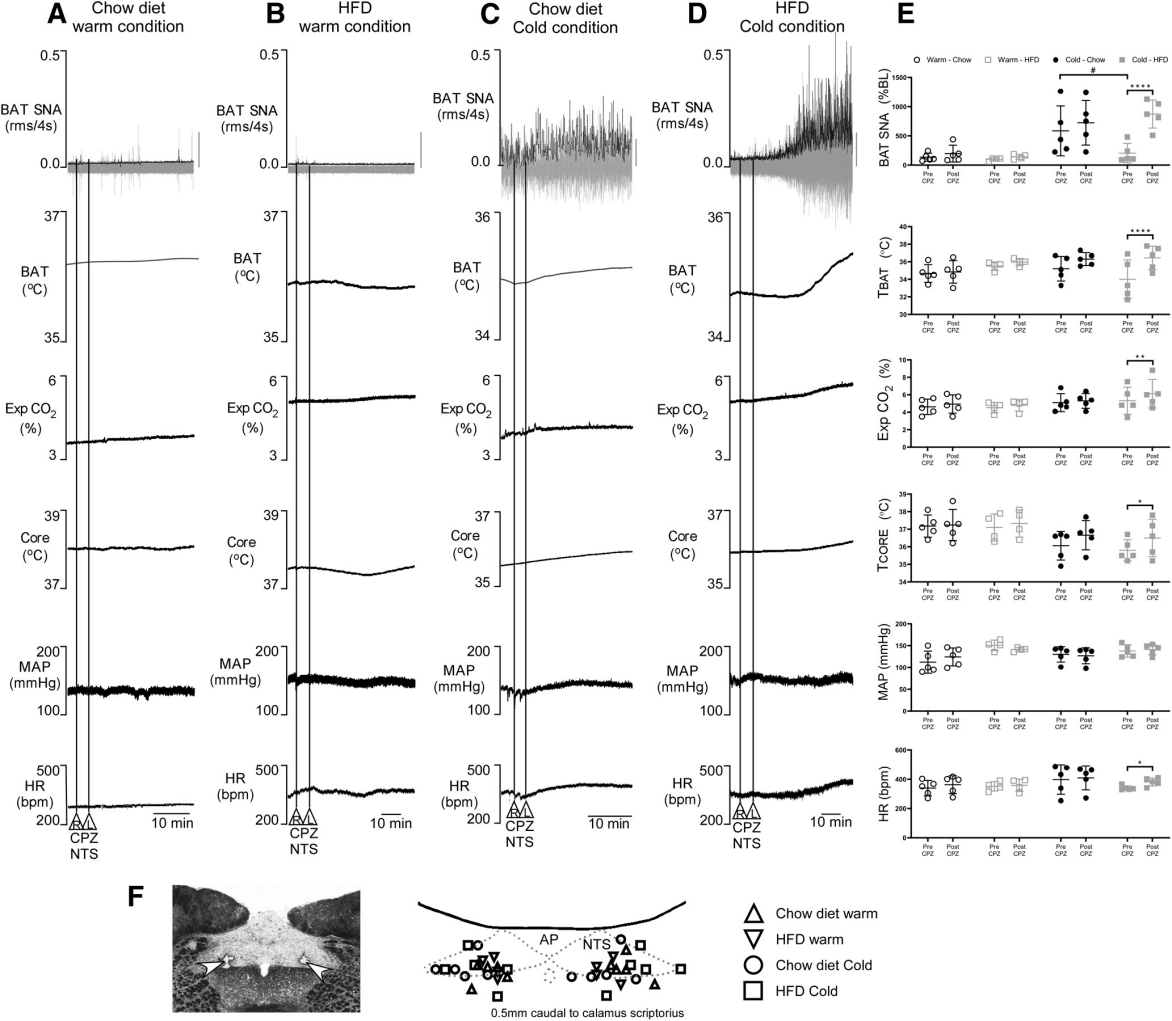
Effects of nanoinjections of CPZ into the mNTS. Representative tracing of BAT SNA, TBAT, TCORE, Exp CO_2_, MAP, and HR of chow (***A***) and HFD (***B***) groups under a warm skin (∼37°C) condition, and of chow (***C***) and HFD (***D***) groups during a cold condition (TSKIN ∼35°C). For BAT SNA, gray vertical bars equivalent to 100 μV. ***E***, Whisker dot plots of individual data, mean (line), and SEM for the physiological variables between pre-CPZ and post-CPZ nanoinjections in chow fed and HFD groups (*n* = 4–5 rats per group); *p < 0.05, **p < 0.005, ****p < 0.0001, #*p* < 0.05 unpaired one-tailed *t* test. ***F***, Representative photomicrograph of partial coronal section illustrating nanoinjection sites in NTS (arrowheads) and schematic representation of all CPZ nanoinjection sites in NTS. AP, area postrema; L, left; R, right.

In the warm condition, with TSKIN and TCORE maintained at ∼37°C, nanoinjection of CPZ into mNTS did not change any of the physiological variables in either chow (Fig. 1*A*,*E*) or HFD (Fig. 1*B*,*E*) rats. During cold exposure (∼35°C TSKIN and TCORE), CPZ in the mNTS did not significantly affect any of the physiological variables in the chow group (Fig. 1*C*,*E*). In contrast, nanoinjection of CPZ into the mNTS of the HFD rats during cold exposure increased BAT SNA (+645 ± 199% BL vs pre-CPZ control [95% confidence interval (95%CI) 4.94e+02, 7.92e+02]; *F*_(1,15)_ = 43.71; *p* < 0.0001), TBAT (+2.4 ± 1.4°C vs pre-CPZ control, [95%CI 1.42, 3.48]; *F*_(1,15)_ = 26.10; *p* < 0.0001), Exp CO_2_ (+1.1 ± 0.9% vs pre-CPZ control, [95%CI 0.26, 1.2]; *p* = 0.003), TCORE (+0.7 ± 0.9°C vs pre-CPZ control; [95%CI 0.2, 1.58]; *F*_(1,15)_ = 9.766; *p* = 0.048) and HR (+42 ± 31 beats/min vs pre-CPZ control; [95%CI 17.0, 63.4]; *F*_(1,15)_ = 8.55; *p* = 0.03; Fig. 1*D*,*E*). MAP was not significantly affected by nanoinjections of CPZ into the mNTS in the HFD group during cold exposure. These results indicate that, in contrast to chow-fed rats, there is a tonic activation of TRPV1 in the mNTS of HFD rats which acts to prevent the normal, cold-evoked activation of BAT SNA.

### Levels of LA metabolites in the plasma and mNTS of chow-fed rats and HFD rats

Since blockade of TRPV1 receptors in the mNTS reversed the HFD-induced impairment of BAT activity (Fig. 1), and the HFD used in this study contains higher levels of LA than the chow diet, we asked whether HFD feeding influences the levels of LA metabolites, some of which are endogenous ligands of TRPV1 (Patwardhan et al., 2010; Osthues and Sisignano, 2019), in the plasma and mNTS. Eicosanoid content in the plasma, mNTS, and DMH were measured by LC-MS analysis (for the complete eicosanoid panel, see Table 1). The DMH (results in Table 1) was sampled as a control region to assess whether the changes observed were widespread or more specific to the mNTS.

**Table 1:** Plasma levels of eicosanoids (ng/ml)

	Chow (mean ± SEM)	HFD (mean ± SEM)	Unpaired mean difference [95%CI]
PGE2	2.41±0.57	2.55±0.77	0.14 [−1.31, 2.19]
13,14dh-15k-PGE2	0.15±0.04	0.15±0.03	0.00 [−0.07, 0.09]
PGD2	1.05±0.2	1.04±0.2	−0.01 [−0.50, 0.49]
13,14dh-15k-PGD2	0.21±0.05	0.19±0.04	−0.2 [−0.13, 0.09]
PGF2α	0.23±0.06	0.21±0.07	−0.2 [−0.15, 0.16]
15-keto PGF2α	0.14±0.03	0.14±0.04	0.0 [−0.08, 0.11]
6,15-diketo PGFα	0.60±0.1	0.8±0.15	0.2 [−0.13,0.50]
TXB2	24.32±4.73	25.57±6.48	1.25 [−11.0, 18.5]
5(S),12(S)-DiHETE	0.47±0.15	0.31±0.07	−0.16 [−0.51, 0.08]
9-HODE	2.48±0.3	1.93±0.28	−0.54 [−0.15, 1.31]
13-HODE	11.15±1.42	7.37±1.11	−3.78 [−7.1, −0.65]
9-HOTrE	0.29±0.04	0.10±0.02	−0.19 [−0.30, −0.11]
13-HOTrE	0.67±0.11	0.08±0.02	−0.59 [−0.81, −0.42]
15-HEDE	0.04±0.005	0.04±0.01	0.00 [−0.02, 0.01]
8-HETrE	0.06±0.01	0.04±0.01	−0.01 [−0.03, 0.01]
5-HETE	1.50±0.13	1.40±0.16	−0.1 [−0.49, 0.26]
8-HETE	0.52±0.07	0.34±0.05	−0.18 [−0.34, −0.03]
11-HETE	8.00±1.56	7.88±2.17	−0.12 [−4.17, 5.55]
12-HETE	45.15±8.09	41.05±10.16	−4.1 [−26.3, 19.9]
15-HETE	1.87±0.29	1.94±0.33	0.07 [−0.68, 0.88]
20-HETE	0.76±0.08	0.85±0.2	0.09 [−0.28, 0.49]
tetranor 12-HETE	0.06±0.01	0.07±0.01	0.01 [−0.01, 0.04]
12-HHTrE	0.44±0.1	0.74±0.2	0.30 [−0.01, 0.82]
5-HEPE	0.47±0.07	0.04±0.01	−0.43 [−0.56, −0.30]
12-HEPE	1.77±0.3	0.23±0.08	−1.54 [−2.18, −1.06]
18-HEPE	0.18±0.02	0.03±0.01	−0.16 [−0.20, −0.11]
4-HDoHE	0.60±0.05	0.22±0.02	−0.38 [−0.48, −0.30]
7-HDoHE	0.22±0.03	0.03±0.01	−0.19 [−0.24, −0.15]
8-HDoHE	0.69±0.05	0.16±0.02	−0.53 [−0.64, −0.43]
10-HDoHE	0.49±0.05	0.09±0.01	−0.40 [−0.50, −0.31]
11-HDoHE	0.33±0.02	0.07±0.01	−0.26 [−0.30, −0.21]
13-HDoHE	0.45±0.04	0.13±0.01	−0.32 [−0.39, −0.25]
14-HDoHE	0.97±0.09	0.42±0.06	−0.55 [−0.71, −0.34]
16-HDoHE	0.30±0.03	0.10±0.01	−0.20 [−0.26, −0.14]
17-HDoHE	0.20±0.01	0.12±0.04	−0.08 [−0.13, 0.02]
20-HDoHE	0.35±0.03	0.09±0.01	−0.26 [−0.31, −0.20]
9(10)-EpOME	10.47±1.39	6.40±1.55	−4.07 [−7.69, −0.30]
12(13)-EpOME	13.40±4.35	3.10±0.43	−10.3 [−21.5, −4.34]
5(6)-EpETrE	1.07±0.16	1.74±0.47	0.67 [−0.19, 1.59]
11(12)-EpETrE	1.26±0.15	1.30±0.31	0.04 [−0.57, 0.66]
14(15)-EpETrE	0.65±0.03	0.74±0.13	0.09 [−0.14, 0.31]
7(8)-EpDPE	0.24±0.04	0.08±0.02	−0.16 [−0.25, −0.08]
10(11)-EpDPE	0.92±0.16	0.32±0.08	−0.60 [−0.93, −0.29]
13(14)-EpDPE	0.41±0.06	0.14±0.03	−0.27 [−0.41, −0.15]
16(17)-EpDPE	0.35±0.03	0.10±0.02	−0.25 [−0.32,−0.19]
19(20)-EpDPE	0.79±0.07	0.16±0.02	−0.63 [−0.77, −0.51]
9,10-DiHOME	6.69±11.16	1.23±0.11	−5.45 [−7.6, −3.43]
12,13-DiHOME	6.89±11.52	0.87±0.16	−6.02 [−9.2, −3.62]
5,6-DiHETrE	0.29±0.05	0.12±0.00	−0.17 [−0.27, −0.09]
8,9-DiHETrE	0.07±0.01	0.05±0.01	−0.02 [−0.03, 0.003]
11,12-DiHETrE	0.97±0.08	0.88±0.07	−0.09 [−0.27, 0.11]
14,15-DiHETrE	1.08±0.13	1.01±0.09	−0.07 [−0.41, 0.17]
19,20-DiHDoPE	1.02±0.09	0.21±0.01	−0.81 [−0.99, −0.67]
9-OxoODE	0.82±0.11	0.74±0.16	−0.08 [−0.38, 0.32]
13-OxoODE	2.59±0.34	1.80±0.36	−0.79 [−1.66, 0.08]
9-OxoOTrE	0.06±0.004	0.04±0.01	−0.02 [−0.03, 0.002]
5-oxoETE	0.18±0.03	0.16±0.02	−0.02 [−0.09, 0.05]
12-OxoETE	0.09±0.02	0.15±0.02	0.06 [0.006, 0.117]

Eicosanoid levels in NTS (ng/mg of tissue)			
	Chow	HFD	Mean difference [95%CI]
PGD2	0.11±0.06	0.16±0.04	0.05 [−0.11, 0.15]
9-HODE	0.06±0.01	0.09±0.01	0.03 [−0.005, 0.058]
13-HODE	0.39±0.06	0.48±0.05	0.09 [−0.09, 0.22]
5-HETE	0.07±0.01	0.10±0.01	0.03 [0.00, 0.06]
8-HETE	0.032±0.01	0.045±0.01	0.013 [−0.001, 0.026]
11-HETE	0.20±0.04	0.26±0.04	0.06 [−0.05, 0.15]
12-HETE	0.33±0.2	0.29±0.1	−0.04 [−0.44, 0.23]
15-HETE	0.12±0.02	0.16±0.02	0.04 [−0.01, 0.09]
20-HETE	0.20±0.04	0.24±0.03	0.04 [−0.06, 0.11]
4-HDoHE	0.026±0.004	0.029±0.002	0.003 [−0.008, 0.009]
8-HDoHE	0.028±0.005	0.034±0.003	0.006 [−0.009, 0.014]
10-HDoHE	0.021±0.003	0.024±0.002	0.003 [−0.008, 0.008]
11-HDoHE	0.025±0.005	0.030±0.002	0.005 [−0.011, 0.012]
13-HDoHE	0.030±0.006	0.033±0.003	0.003 [−0.014, 0.012]
14-HDoHE	0.027±0.007	0.029±0.002	0.002 [−0.019, 0.010]
16-HDoHE	0.040±0.005	0.046±0.002	0.006 [−0.01, 0.01]
20-HDoHE	0.035±0.006	0.038±0.003	0.003 [−0.014, 0.011]
9(10)-EpOME	0.267±0.04	0.53±0.1	0.266 [0.049, 0.429]
12(13)-EpOME	0.194±0.03	0.36±0.1	0.163 [0.013, 0.276]
5(6)-EpETrE	0.208±0.05	0.42±0.1	0.212 [0.064, 0.345]
11(12)-EpETrE	0.587±0.13	0.67±0.1	0.083 [−0.265, 0.355]
14(15)-EpETrE	0.333±0.07	0.39±0.06	0.057 [−0.149, 0.201]
7(8)-EpDPE	0.031±0.006	0.030±0.003	−0.001 [−0.017, 0.009]
10(11)-EpDPE	0.182±0.04	0.18±0.02	−0.002 [−0.124, 0.066]
13(14)-EpDPE	0.106±0.02	0.10±0.01	−0.006 [−0.064, 0.033]
16(17)-EpDPE	0.071±0.01	0.07±0.01	−0.001 [−0.041, 0.021]
9,10-DiHOME	0.048±0.01	0.10±0.02	0.052 [0.017, 0.106]
12,13-DiHOME	0.047±0.01	0.11±0.02	0.066 [0.025, 0.114]
9-OxoODE	0.045±0.01	0.06±0.01	0.015 [−0.019, 0.034]
13-OxoODE	0.120±0.03	0.18±0.03	0.06 [−0.021, 0.132]

Eicosanoid levels in DMH (ng/mg of tissue)		
	Chow	HFD	Mean difference [95%CI]
PGE2	0.072±0.012	0.119±0.013	−0.047 [−0.076, −0.014]
PGD2	0.082±0.019	0.197±0.040	−0.115 [−0.198, −0.039]
PGF2α	0.037±0.012	0.073±0.019	−0.036 [−0.083, −0.001]
TXB2	0.057±0.017	0.121±0.031	−0.064 [−0.135, −0.005]
9-HODE	0.058±0.009	0.081±0.038	−0.023 [−0.140, 0.024]
13-HODE	0.348±0.061	0.403±0.120	−0.055 [−0.388, 0.132]
5-HETE	0.059±0.010	0.104±0.018	−0.045 [−0.09, −0.013]
8-HETE	0.022±0.003	0.035±0.006	−0.013 [−0.033, −0.004]
11-HETE	0.156±0.024	0.271±0.037	−0.115 [−0.20, −0.042]
12-HETE	0.056±0.009	0.074±0.011	−0.018 [−0.047, 0.005]
15-HETE	0.096±0.011	0.145±0.016	−0.049 [−0.09,−0.019]
20-HETE	0.088±0.022	0.132±0.025	−0.044 [−0.106, 0.013]
12-HHTrE	0.016±0.003	0.032±0.004	−0.016 [−0.025, −0.008]
4-HDoHE	0.012±0.003	0.023±0.004	−0.011 [−0.021, −0.002]
10-HDoHE	0.009±0.001	0.010±0.001	−0.001 [−0.005, 0.002]
11-HDoHE	0.009±0.002	0.013±0.002	−0.004 [−0.009, 0.001]
13-HDoHE	0.010±0.002	0.015±0.002	−0.005 [−0.01, 0.001]
16-HDoHE	0.015±0.002	0.019±0.003	−0.004 [−0.011, 0.002]
12(13)-EpOME	0.085±0.015	0.096±0.017	−0.011 [−0.050, 0.031]
5(6)-EpETrE	0.121±0.033	0.156±0.031	−0.035 [−0.126, 0.041]
11(12)-EpETrE	0.285±0.080	0.420±0.095	−0.135 [−0.357, 0.088]
14(15)-EpETrE	0.155±0.046	0.217±0.049	−0.062 [−0.179, 0.065]
7(8)-EpDPE	0.013±0.004	0.022±0.005	−0.009 [−0.022, 0.001]
10(11)-EpDPE	0.051±0.014	0.080±0.021	−0.029 [−0.081, 0.012]
13(14)-EpDPE	0.028±0.007	0.040±0.010	−0.012 [−0.033, 0.011]
16(17)-EpDPE	0.019±0.005	0.027±0.007	−0.008 [−0.023, 0.007]
9,10-DiHOME	0.040±0.009	0.044±0.013	−0.004 [−0.038, 0.021]
9-OxoODE	0.038±0.006	0.055±0.021	−0.017 [−0.08, 0.009]
13-OxoODE	0.094±0.016	0.155±0.076	−0.061 [−0.294, 0.031]
5-oxoETE	0.020±0.003	0.039±0.008	−0.019 [−0.035, −0.003]

**Figure 2.**
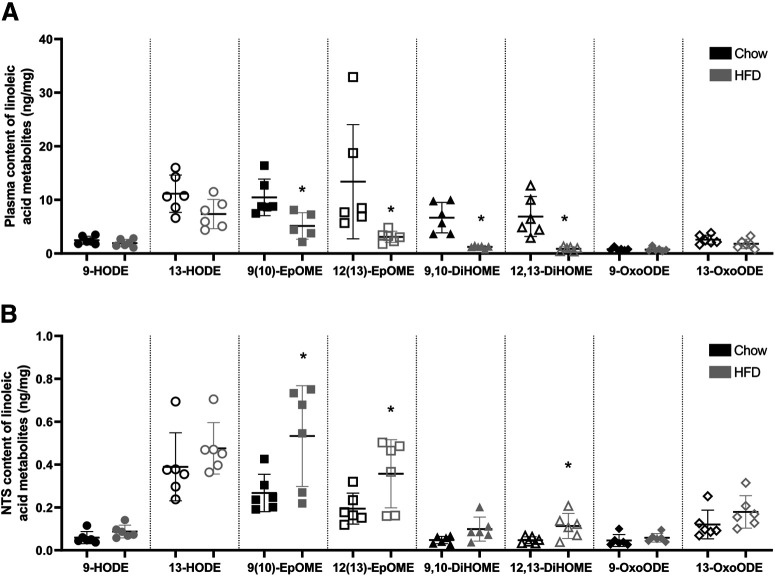
Content of LA metabolites. Levels in (***A***) plasma and (***B***) the mNTS of chow-fed rats (black symbols) and HFD-fed rats (gray symbols). The concentrations of 9-HODE (filled circles), 13-HODE (empty circles), 9(10)-EpOME (filled squares), 12(13)-EpOME (empty squares), 9,10-DiHOME (filled triangles), 12,13-DiHOME (empty triangles), 9-OxoODE (filled diamonds), and 13-OxoODE (empty diamonds) are expressed as mean ± SEM (*n* = 6 rats per group); * HFD versus chow, *p* < 0.05, two-sided permutation *t* test.

Compared with chow-fed rats, the HFD rats had lower plasma levels of several metabolites of LA: 12(13)-EpOME (mean difference between chow and HFD is 10.3 ng/ml, *p* = 0.000), 9(10)-DiHOME (mean difference between chow and HFD is 5.45 ng/ml, *p* = 0.000), and 12(13)-DiHOME (mean difference between chow and HFD is 6.02 ng/ml, *p* = 0.0006; Fig. 2*A*; Table 1). The plasma levels of 9-HODE, 13-HODE, 9(10)-EpOME, 9-OxoODE, and 13-OxoODE were not significantly different between the groups (Fig. 2*A*; Table 1).

Compared with chow-fed rats, the HFD rats had higher levels of several LA metabolites in the mNTS: 9(10)-EpOME (mean difference between chow and HFD is 0.266 ng/mg, *p* = 0.0318), 12(13)-EpOME (mean difference between chow and HFD is 0.163 ng/mg, *p* = 0.0498), 9(10)-DiHOME (mean difference between HFD and chow-fed is 0.052 ng/mg, *p* = 0.0244), and 12(13)-DiHOME (mean difference between HFD and chow-fed is 0.0662 ng/mg, *p* = 0.017; Fig. 2*B*). The mNTS content of 9-HODE, 13-HODE, 9-OxoODE, and 13-OxoODE were not significantly different between the groups (Fig. 2*B*; Table 1). In the DMH, the levels of LA metabolites were not different between chow-fed and HFD-fed rats (Table 1). These results are consistent with the potential for metabolites of dietary LA to tonically stimulate TRPV1 in the mNTS and reduce BAT thermogenesis and energy expenditure in HFD-fed rats in a subthermoneutral environment.

### Functional neuroanatomy: mNTS-LPBd-MnPO pathway

**Figure 3.**
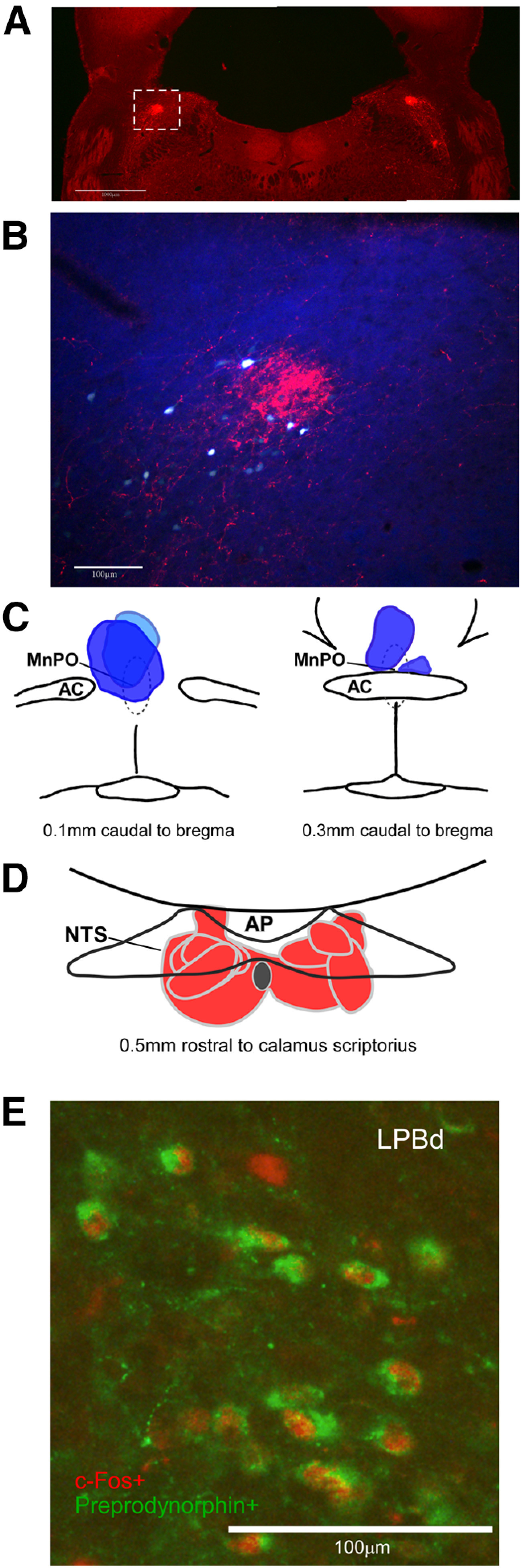
Anatomical connections between the NTS, LPBd, and MnPO, as well as c-Fos expression in ppDyn neurons in the LPBd following cold exposure. ***A***, Representative photomicrograph of a partial coronal section through the LPB illustrating the location of BDA-labeled boutons and varicose axons in this region (red) following nanoinjection of BDA in the NTS. Scale bar: 1 mm. ***B***, Higher power photomicrograph of the section within the white box in panel ***A*** showing BDA-labeled boutons and varicose axons (red) in the LPBd following nanoinjection of BDA in the NTS, and retrogradely labeled cell bodies (white/blue) in the LPBd following FG nanoinjections in the MnPO, many retrogradely-labeled neurons had at least one close apposition from a BDA-labeled bouton or varicose axon, similar data were found in four rats. Scale bar: 100 μm. ***C***, Schematic representation of FG injection sites into MnPO (from −0.1 and −0.3 mm caudal to bregma). AC, anterior commissure. ***D***, Schematic representation of BDA injection sites into NTS (−0.5 mm rostral to calamus). AP, area postrema. ***E***, Representative photomicrograph of a partial coronal section showing immunohistochemically-labeled c-Fos (red) following cold exposure predominately in ppDyn positive neurons (green) in the LPBd of a HFD rat. Scale bar: 100 μm.

To investigate potential BAT sympathoinhibitory circuits driven by neurons in the mNTS, we nanoinjected the anterograde tracer, BDA, in the mNTS (Fig. 3*D*) to map the projections of mNTS neurons. In agreement with earlier studies (Ricardo and Koh, 1978; Herbert et al., 1990; Ruggiero et al., 1998; Geerling and Loewy, 2006), BDA-labeled axons from mNTS neurons were found in the LPB (Fig. 3*A*), as well as in the ventral lateral medulla, the amygdala, the ventrolateral periaqueductal gray, the hypothalamus, the bed nucleus of the stria terminalis, and the paraventricular thalamus. Since LPBd neurons projecting to the MnPO can inhibit BAT activity (Nakamura and Morrison, 2010; Yang et al., 2020; Norris et al., 2021), we assessed the association of axons and boutons from mNTS with LPBd neurons retrogradely-labeled following nanoinjection of the retrograde tracer, FG, in the MnPO (Fig. 3*C*). BDA-labeled axon terminals of mNTS neurons were in close proximity to the processes of LPBd neurons that were retrogradely-labeled following FG nanoinjections in MnPO (Fig. 3*A*,*B*). The LPBd contains dynorphinergic neurons with projections to the MnPO, therefore to determine whether the neurons in the LPBd that were active during cooling were dynorphinergic, we labeled tissue sections through the LPBd of cold-exposed rats for ppDyn and c-fos. Many of the neurons in the LPBd that expressed c-fos following cold exposure were ppDyn positive (Fig. 3*E*). In separate tissue sections, we assessed whether LPBd neurons that project to MnPO express c-fos following cold exposure. In HFD rats compared with chow-fed rats, a higher proportion of LPBd neurons that were retrogradely-labeled following FG nanoinjection in MnPO expressed c-fos following cold exposure (17 ± 6% vs 6 ± 1%, respectively; mean difference 10.8 [95%CI 0.75, 20.0]; two-sided permutation *t* test, *p* = 0.0312). These results are consistent with a neural pathway connecting the mNTS and the MnPO through the LPBd, that could mediate the inhibition of BAT activity following activation of mNTS neurons (Cao et al., 2010) or exposure to a HFD (Madden and Morrison, 2016).

### Blockade of KOR in the MnPO recovers the cold-evoked BAT thermogenic response in HFD rats

**Figure 4.**
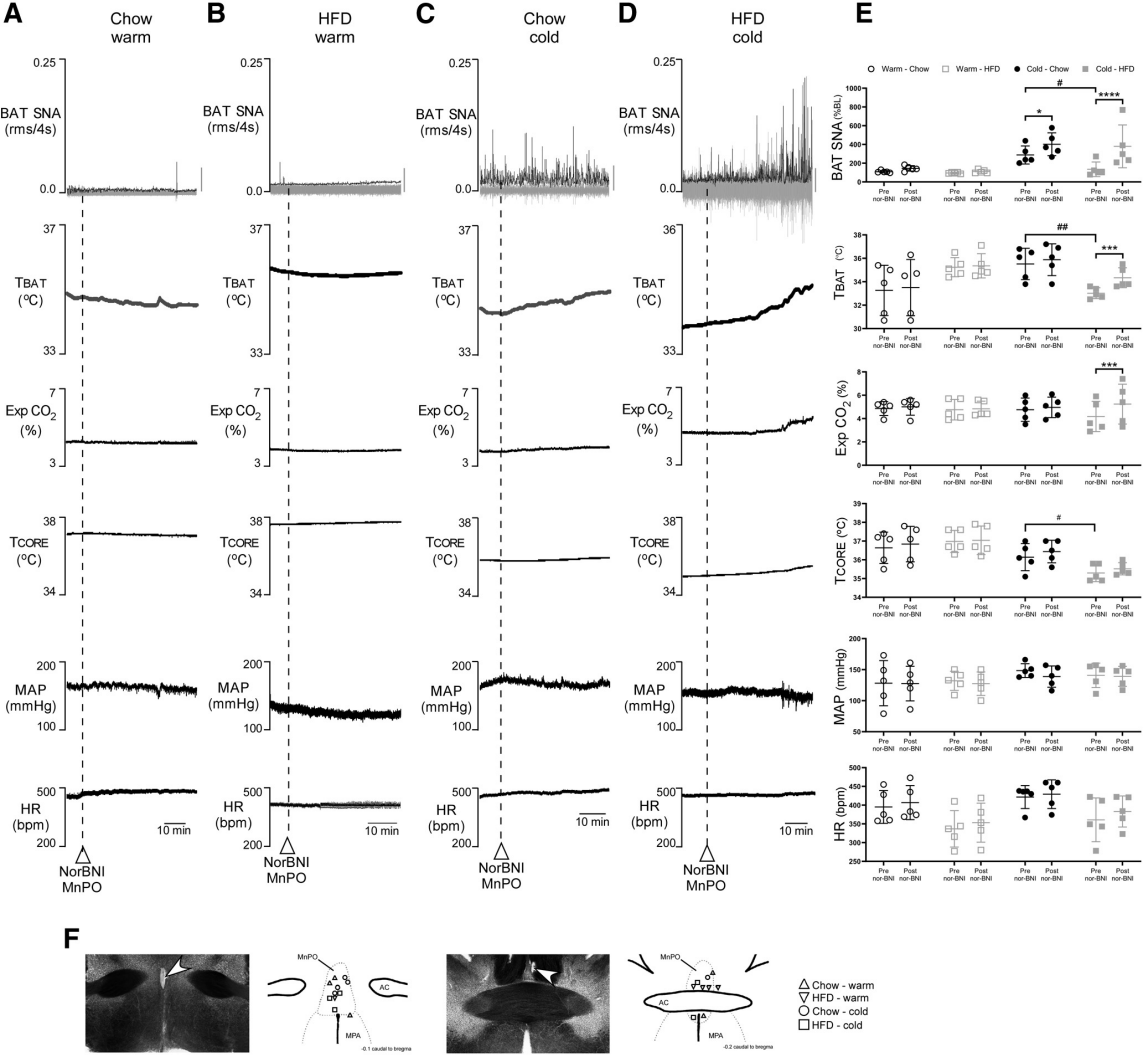
Effects of nanoinjections of nor-BNI dihydrochloride into the MnPO. Representative tracing of BAT SNA, TBAT, TCORE, Exp CO_2_, MAP, and HR of chow (***A***) and HFD (***B***) groups under a warm condition (TSKIN and TCORE ∼37°C), and of chow (***C***) and HFD (***D***) groups under a cold condition (TSKIN and TCORE ∼35°C). For BAT SNA gray vertical bars equivalent to 100 μV. ***E***, Whisker dot plots of individual data, mean (line) and SEM for the physiological variables between pre-nor-BNI and post-nor-BNI nanoinjections in chow fed and HFD rats; *p < 0.05, ***p < 0.001, ****p < 0.0001, three-way ANOVA, #*p* < 0.05 unpaired one-tailed *t* test, ##*p* < 0.005, unpaired one-tailed *t* test. ***F***, Schematic representation of nor-BNI nanoinjection sites into MnPO (*n* = 5 rats per group). AC, anterior commissure.

ppDyn neurons play a role in decreasing BAT thermogenesis and in energy homeostasis during HFD (Yang et al., 2020) and KOR activation in the MnPO induces hypothermia (Xin et al., 1997). Thus, we tested the hypothesis that KOR activation in MnPO contributes to the impairment of cold-evoked BAT thermogenesis in HFD rats. As previously reported (Madden and Morrison, 2016), the level of cold-evoked BAT SNA and TBAT was lower in HFD rats compared with chow-fed rats (Fig. 4*E*). However, during cold exposure, nanoinjection of the KOR antagonist, nor-BNI, into the MnPO of HFD rats increased their BAT SNA (+244 ± 152% BL vs pre-nor-BNI; *F*_(1,16)_ = 31.63; *p* < 0.0001), TBAT (+1.3 ± 0.5°C vs pre-nor-BNI; *F*_(1,16)_ = 15.13; *p* = 0.0005), and Exp CO_2_ (+1.1 ± 0.8% vs pre-nor-BNI; *F*_(1,16)_ = 14.84; *p* = 0.0002; Fig. 4*D*,*E*) to levels not different from those in chow-fed rats experiencing the same cold exposure. The TCORE, MAP, and HR in all rats were unaffected by nanoinjections of nor-BNI into MnPO. During cold exposure, nanoinjection of nor-BNI into MnPO of chow-fed rats also increased BAT SNA (+114.4 ± 48.4% BL vs pre-nor-BNI, *p* = 0.025; Fig. 4*C*,*E*). During the warm condition (TSKIN and TCORE at ∼37°C), nanoinjection of nor-BNI into MnPO did not change the physiological variables in the chow-fed (Fig. 4*A*,*E*) or the HFD (Fig. 4*B*,*E*) groups.

**Figure 5.**
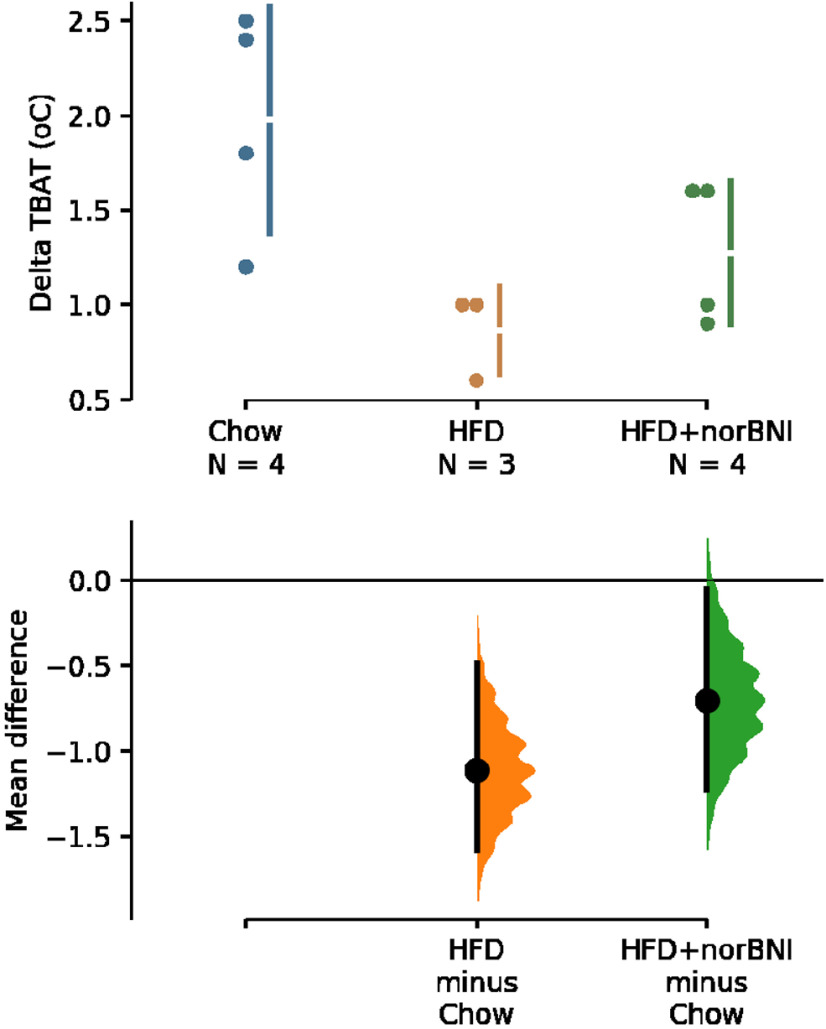
Effect of systemic nor-BNI dihydrochloride on HFD-induced impairment of cold-evoked BAT thermogenesis in free-behaving rats. A Cummings estimation plot illustrating the cold-evoked change in TBAT for HFD rats and HFD rats receiving an intraperitoneal injection of nor-BNI before cold exposure, each compared against the shared control chow-fed group. The raw change in TBAT is plotted on the upper axes. On the lower axes, mean differences are plotted as bootstrap sampling distributions. Each mean difference is depicted as a dot. Each 95% confidence interval is indicated by the ends of the vertical error bars.

In parallel experiments in unanesthetized, free-behaving rats, cold exposure (ambient temperature: 15°C) increased TBAT by a maximum of +2.0 ± 0.3°C from a BL of 36.6 ± 0.4°C in chow-fed rats compared with +0.9 ± 0.1°C from a BL of 36.4 ± 0.3°C in HFD rats and +1.3 ± 0.2°C from a BL of 36.3 ± 0.4°C in HFD rats pretreated intraperitoneally with nor-BNI (Fig. 5). The unpaired mean difference between chow and HFD was −1.11 [95.0%CI −1.58, −0.483] (*p* = 0.0472, two-sided permutation *t* test). The unpaired mean difference between chow and HFD rats receiving nor-BNI was −0.7 [95.0%CI −1.23, −0.05] (*p* = 0.0882, two-sided permutation *t* test).

## Discussion

To date, there has been no explanation for the consistent observation that BAT FDG uptake, an indirect measure of BAT thermogenesis and BAT energy expenditure, is significantly reduced in cold-exposed, obese adult humans (Cypess et al., 2009; Saito et al., 2009; van Marken Lichtenbelt et al., 2009; Orava et al., 2013). We present evidence supporting a novel neural pathway mediating the impairment of sympathetically-mediated BAT thermogenesis and BAT metabolism in rats after prolonged exposure to a HFD. Pharmacological blockade of TRPV1 in the NTS recovered the BAT thermogenic response to cold exposure in HFD rats and chronic HFD increased the content of endogenous ligands for TRPV1 in the mNTS. These data indicate that TRPV1 activation in the mNTS is necessary for the reduced BAT activation in HFD rats, and implicate TRPV1-stimulating metabolites of dietary lipid in driving a mNTS efferent pathway that inhibits cold-evoked BAT thermogenesis. Blockade of KORs in the MnPO also reversed the HFD-induced impairment of cold-evoked BAT SNA and BAT thermogenesis, indicating that a dynorphinergic input to the MnPO is necessary for the HFD-induced impairment of BAT activation. Our demonstration of close appositions between the axon terminals of mNTS neurons and the processes of LPBd neurons that project to the MnPO reveals a potential anatomic substrate for an mNTS–LPBd–MnPO pathway through which increased TRPV1 activation in mNTS results in increased dynorphin release and KOR activation in MnPO. Together these observations support a model in which maintenance on a HFD leads to elevated levels of TRPV1 ligands in the mNTS, which activates TRPV1 on the terminals of vagal afferents in the mNTS, leading to a glutamatergic increase in the discharge of BAT sympathoinhibitory neurons in the mNTS that project to LPBd. We propose that the mNTS input excites LPBd dynorphinergic neurons whose terminals release dynorphin in the MnPO area, potentially onto the BAT sympathoexcitatory neurons necessary of cold-evoked activation of BAT (da Conceicao et al., 2020), resulting in a KOR-mediated attenuation of the cold-evoked activation of BAT SNA, thereby reducing BAT thermogenic energy expenditure.

The mNTS plays a significant role in the regulation of the sympathetic and parasympathetic outflows to the cardiovascular system (Andresen and Kunze, 1994). In addition, LPBd dynorphinergic and glutamatergic neurons with projections to the MnPO play an essential role in the warm-defensive inhibition of cutaneous vasoconstriction (Nakamura and Morrison, 2010; Norris et al., 2021). It remains an open question whether the HFD-induced increase in the activity of mNTS neurons projecting to LPBd also influences other thermoeffector responses to cold, such as cutaneous vasoconstriction and shivering, or other cardiovascular sympathetic pathways contributing to HFD-induced hypertension.

LA is the most abundant polyunsaturated fatty acid consumed in the United States diet (Blasbalg et al., 2011). Thus, feeding the HFD rats in our study with a diet that has a 10-fold higher concentration of LA than the chow diet fed to the control group may mimic the exposure to LA that humans consuming fatty, processed foods would receive. Our finding that several oxidized products of LA, including 9(10)-EpOME, 12(13)-EpOME, 9(10)-DiHOME, and 12(13)-DiHOME, are increased in the mNTS of the HFD rats compared with chow-fed rats, while the plasma levels of these same LA metabolites were reduced, suggests that the elevation of LA products in the mNTS results from increased local production, decreased degradation, and/or a binding enhancement within the mNTS. The exact mechanism(s) that leads to the accumulation of oxidized LA metabolites in the mNTS versus plasma, whether their presence in the mNTS affects neurotransmission in the proposed BAT sympathoinhibitory pathway, and whether high fat and/or obesogenic diets lacking elevated levels of LA also induce an impairment of the cold-induced sympathetic activation of BAT remain to be determined.

Oxidized products of LA, such as 9(10)-EpOME, 12(13)-EpOME, 9(10)-DiHOME, and 12(13)-DiHOME are endogenous agonists for TRPV1 (Green et al., 2016) and both the central and peripheral actions of TRPV1 agonists influence energy homeostasis. In the context of thermoregulation, the peripheral actions of TRPV1 agonists, such as capsinoids (Nirengi et al., 2016) and 12(13)-diHOME (Lynes et al., 2017), are thermogenic. In contrast, the activation of the TRPV1 channels in vagal nerve terminals enhances the release of glutamate in the mNTS (Doyle et al., 2002), which inhibits BAT SNA and decreases BAT thermogenesis (Mohammed et al., 2018). Thus, both the elevated levels of LA metabolites that we observed in the mNTS of HFD rats, combined with the lower levels of peripheral TRPV1 agonists in HFD rats compared with chow-fed rats, would contribute to conditions favoring decreased BAT thermogenesis and metabolism, thereby contributing to weight gain on an HFD.

Our finding that CPZ blockade of TRPV1 in the mNTS of the HFD rats increased BAT SNA and thermogenesis during cold exposure, coupled with our demonstration that nanoinjections of CPZ into the mNTS blocks the sympathoinhibitory effects produced by nanoinjections in the mNTS of the TRPV1 agonist, resiniferatoxin (Mohammed et al., 2018), indicates that TRPV1 must be continuously activated to maintain the reduction in cold-defensive BAT activation we observe in HFD rats. Endogenous lipid metabolites such as those that were elevated in the mNTS of HFD rats (Fig. 2) would be candidate molecules for such activation of TRPV1 in the mNTS of HFD rats. The dorsal motor nucleus of the vagus (DMV) is in close proximity to the mNTS and TRPV1 are present in the DMV. However, since BAT receives no input from DMV neurons (Cano et al., 2003), it seems unlikely that TRPV1 inhibition in the DMV played any role in the effects of CPZ on the cold-evoked BAT SNA and BAT thermogenesis in our experiments. Rather, TRPV1 on the terminals of vagal afferents in mNTS (Hermes et al., 2016) are likely to be the relevant TRPV1 for the effects of both endogenous lipid metabolites in mNTS, and the CPZ we injected into the mNTS, since TRPV1 agonists potentiate vagal afferent inhibition of BAT SNA (Mohammed et al., 2018) and vagal afferent input is absolutely required for the impairment of cold-induced BAT activation in HFD rats (Madden and Morrison, 2016). We propose that the targets of the relevant vagal terminals are second-order, vagal sensory neurons (Doyle et al., 2002; Hofmann and Andresen, 2016) in mNTS whose activation drives a reduction in BAT SNA (Cao et al., 2010; Madden et al., 2017) that is mediated via a mNTS projection to the LPBd.

In warm rats, neither the blockade of TRPV1 in the mNTS, nor the blockade of KORs in MnPO, altered the measured physiological variables in either diet group. The most likely explanation for this observation is that there is an inadequate underlying sympathoexcitatory thermogenic drive under the warm condition. In particular, in the face of the inhibition of the BAT sympathoexcitatory neurons in the MnPO (da Conceicao et al., 2020) by cutaneous warm thermoreceptors it appears to be inconsequential whether blockade of TRPV1 in the mNTS or KORs in MnPO removes the tonic BAT sympathoinhibitory drive present in HFD rats. Indeed, the absence of a BAT activation following nor-BNI injection in the MnPO of warm rats suggests that the warming-evoked inhibition of BAT SNA is not dependent on a dynorphin input to MnPO. The interaction between warming-activated and mNTS-activated LPBd neuronal projections to MnPO awaits further investigation.

Medial NTS neurons project to several brain areas that play a role in thermoregulation (Shafton et al., 1999; Vrang et al., 2007; Affleck et al., 2012; Roman et al., 2017). In agreement with earlier tracing studies (Geerling and Loewy, 2006; Herbert et al., 1990; Ricardo and Koh, 1978), we also found mNTS neurons that project to the region of the LPBd that contains BAT sympathoinhibitory neurons that innervate the MnPO (Nakamura and Morrison, 2010; Norris et al., 2021) . The neurochemical identity of the relevant mNTS innervation of the LPB remains to be detemined among catecholamines, endomorphin, galanin, cholecystokinin, and corticotropin-releasing factor (Herbert and Saper, 1990; Lü et al., 2009). We also identified MnPO-projecting neurons in the same region of the LPBd that receives close appositions from axon terminals of mNTS neurons. Together with our observation that activation of KORs in the MnPO is necessary for the HFD-induced impairment of cold-evoked BAT SNA and BAT thermogenesis, these data provide strong anatomic support for a model in which mNTS neurons drive release of dynorphin in the MnPO via their activation of MnPO-projecting neurons in the LPBd. How dynorphin influences BAT regulation via neurons in the MnPO remains unknown, but dynorphin’s ability to block glutamate transmission (Crowley et al., 2016) suggests that it could act to interupt the cold-responsive, glutamatergic transmission from neurons in the LPBel to potential GABA neurons in the MnPO (Nakamura and Morrison, 2008a,b). Alternatively, dynorphinergic inputs could block cold-evoked BAT SNA by inhibiting BAT sympathoexcitatory neurons in MnPO which excite BAT thermogenesis-promoting neurons in the DMH whose activity is required for cold-evoked BAT activation (da Conceicao et al., 2020).

The current study describes mechanisms that contribute to a reduction in energy utilization associated with consumption of HFD in rats. Laboratory rodents are normally maintained in the subthermoneutral environment of a vivarium with a temperature of 22–24°C, which represents a chronic cold exposure that will induce BAT thermogenesis and increase metabolic rate by up to 120% (Abreu-Vieira et al., 2015). Thus, any reduction in their cold-evoked BAT activation, such as that we describe here in response to a HFD, will reduce their overall energy expenditure and exacerbate weight gain and dysregulation of blood glucose. Interestingly, one currently FDA-approved weight loss drug, Contrave, contains naltrexone, an opioid receptor antagonist which also decreases food intake (Billes et al., 2014). Our results suggest that naltrexone may also act to increase BAT energy expenditure by reducing the HFD-induced blockade of cold-evoked BAT thermogenesis, which would contribute to its therapeutic efficacy. Increased understanding of the neural pathways affected by HFD and the underlying mechanisms of dysregulation during the pathogenesis of obesity will present additional targets for the development of therapies for obesity and related co-morbidities, such as diabetes.

## Synthesis

Reviewing Editor: Quentin Pittman, University of Calgary Decisions are customarily a result of the Reviewing Editor and the peer reviewers coming together and discussing their recommendations until a consensus is reached. When revisions are invited, a fact-based synthesis statement explaining their decision and outlining what is needed to prepare a revision will be listed below. The following reviewer(s) agreed to reveal their identity: NONE. Note: If this manuscript was transferred from JNeurosci and a decision was made to accept the manuscript without peer review, a brief statement to this effect will instead be what is listed below.

The authors have adequately addressed the reviewers’ comments from the JNS submission. Some additional data have been added and additional discussion incorporated.
